# Associations of cerebrospinal fluid metal ion profiles with depressive symptoms in active smokers: a cross-sectional network analysis

**DOI:** 10.3389/fpsyt.2026.1907670

**Published:** 2026-09-15

**Authors:** Yuying Li, Yuyu Wu, Lingling Chen, Tengyue Chen, Xuan Hu, Chaoyi Guo, Xingguang Luo, Yimin Kang, Li Chen, Yanlong Liu, Fan Wang, Yu-Hsin Chen

**Affiliations:** 1 Ruian People’s Hospital, Wenzhou Medical University Affiliated Third Hospital, Wenzhou, China; 2 School of Medicine, Zhejiang University, Hangzhou, China; 3 School of Mental Health, Wenzhou Medical University, Wenzhou, China; 4 Department of Psychiatry, Yale University School of Medicine, New Haven, CT, United States; 5 Psychosomatic Medicine Research Division, Inner Mongolia Medical University, Huhhot, China; 6 Beijing Hui-Long-Guan Hospital, Peking University, Beijing, China

**Keywords:** cerebrospinal fluid, depression, iron, metal ions, smoking

## Abstract

**Background:**

Smokers are more likely to report depressive symptoms, on the other hand, individuals with depression are also more likely to initiate smoking. In a recent study, higher CSF concentration of magnesium, zinc, iron, lead, lithium, and aluminum were reported among active smokers. This study investigated whether the disruption of central metal homeostasis in active smokers is associated with self-reports of depressive symptoms.

**Methods:**

CSF samples from 161 Chinese male patients who underwent anterior cruciate ligament reconstruction surgical operation following knee joint injuries were obtained. Among the 161 male patients, 57 male patients were active smokers while 104 patients reported never engaged in any form of smoking. Depressive symptoms were measured via Beck Depression Inventory (BDI) self-reports. CSF concentrations of 8 metal ions were quantified by atomic absorption spectrophotometry. Group comparisons, correlation analyses, mixed graphical model network analyses, and moderation models were performed to examine associations among smoking, CSF metal ions, and depressive symptoms.

**Results:**

Active smokers had higher BDI total scores and higher CSF concentrations of magnesium, zinc, iron, lead, lithium, and aluminum than non-smokers. Higher BDI total scores were associated with higher CSF concentration of zinc, iron, lead, and aluminum. Network analysis revealed iron, zinc, and lead ranked the highest in terms of strength, expected influence, and closeness among the 8 metal ions. With iron as an important association node among the three in the metal ion network observed, simultaneously significantly associated with BDI total scores. Further analyses revealed smoking was positively associated with depressive symptoms, and the interaction between smoking and iron significantly predicted BDI total scores. Additional analyses revealed that concentrations of iron in CSF also significantly interacted with zinc and lead.

**Conclusions:**

Iron is an important associated node that connects various other metal ions to self-reports of depressive symptoms. This finding shed light upon the interrelationships among smoking status, depressive symptoms, and metal ions in CSF, providing partial evidence supporting the relevance of central metal dysregulation to the smoking–depressive symptoms among male smokers.

## 1 Introduction

Tobacco smoking and depressive symptoms are major public health concerns that frequently co-occur in both clinical and community populations (1–3). Smokers are more likely to report various degrees of depressive symptoms, whereas individuals with depression are also more likely to initiate smoking, maintain nicotine dependence, and experience greater difficulty quitting smoking (4, 5). This bidirectional association suggests that the relationship between smoking and depressive symptoms extends beyond behavioral comorbidity and may perhaps involve shared biological correlates or pathways. To date, the biological correlates underlying this relationship remain partially understood, particularly at the level of the central nervous system.

One plausible biological pathway involves disruption of metal ion homeostasis. Essential metal ions, including iron, zinc, magnesium, and calcium, play critical roles in neurotransmitter synthesis, mitochondrial function, synaptic activity, myelination, and redox homeostasis. Iron homeostasis is especially tightly regulated through coordinated mechanisms governing absorption, transport, storage, and cellular availability, and disruption of these processes may promote oxidative stress and neurobiological dysfunction (6–8). Potentially neurotoxic metals such as lead and aluminum may also contribute to neuroinflammation, oxidative stress, and neuronal dysfunction. Cigarette smoke contains numerous metal constituents and may alter metal absorption, transport, and systemic distribution, thereby contributing to disturbances in metal homeostasis (3, 9, 10). Smoking-derived or smoking-regulated metals may influence the central nervous system through several non-mutually exclusive pathways, including systemic exposure to inhaled metals, oxidative stress, inflammatory signaling, altered blood-brain barrier permeability, and regulation of metal transporters (11, 12). These processes may affect central metal distribution and metal-metal interactions, thereby influencing neurotransmission, mitochondrial function, redox balance, and neuroinflammatory responses (13, 14). Such disturbances may be particularly relevant to individual’s self-report of depressive symptoms, given the established roles of metal ions in monoaminergic signaling, neuroplasticity, and inflammatory regulation (11, 15–17). To date, the majority of the findings reported were based on peripheral specimens, including blood, urine, or hair, which may not adequately reflect the biochemical environment of the central nervous system (11, 18). Metal ions in the cerebrospinal fluid (CSF), on the other hand, may provide a more biologically informative measure of central metal ion homeostasis.

Accumulating evidence suggests that altered levels of several metals are associated with depressive symptoms and other adverse neuropsychiatric outcomes, although the available findings remain heterogeneous and, in some cases, inconsistent (19–21). Few studies, however, have specifically examined the relationship between CSF metal ion profiles and depressive symptoms in the context of smoking (5, 22). This gap is particularly important because smoking may influence both metal exposure and the biological consequences of metal dysregulation (23). Previous studies have evaluated individual metals in isolation but given how metal ions operate within a highly interconnected biological system, where correlations, co-regulation, and interaction effects may collectively influence neuropsychiatric outcomes (24–28). A network-based analytical framework approach may therefore help identify central or bridging nodes that play disproportionately important roles in the association between smoking-related metal dysregulation and depressive symptoms.

To address these gaps in research, the present study explored CSF metal ion profiles in smokers and non-smokers and examined their associations with various degrees of depressive symptoms assessed by the Beck Depression Inventory (BDI). Demographic characteristics, clinical variables, and CSF metal ion concentrations comparisons between smoking and non-smoking groups were conducted. Correlational analyses between individual CSF metal ions and BDI total scores as well as network analysis were then employed to depict the interrelationships among smoking status, depressive symptoms, and metal ions in CSF. Finally, regression analyses were conducted to examine whether key CSF metal ions moderated the association between smoking and depressive symptoms. We hypothesized that smoking would be associated with altered CSF metal ion profiles, with specific metal ions related to depressive symptom burden. Moreover identify which metal ions would emerge as central nodes within the CSF metal ion network accounting for the largest number of direct or indirect connections with depressive symptoms.

## 2 Materials and methods

### 2.1 Participants

The participant sample examined in this study is a subset sample of a cohort dataset from a previous study (23). The current study examines the data of 161 Chinese males scheduled for anterior cruciate ligament (ACL) reconstruction surgery participated. Only male participants were included in this study, as the surgical cohort contained little to no active female smokers. Among the 161 male patients, 57 were active smokers, and 104 reported never engaging in any form of smoking activity. Sociodemographic data of them, including age, years of education, and body mass index (BMI), were recorded. Clinical data were obtained through self-reporting and verified by family members.

Exclusion criteria included:

1. A family history of psychosis or neurological diseases.
2. The presence of systemic or central nervous system (CNS) diseases, as determined by the Mini International Neuropsychiatric Interview.

Non-smokers were defined as individuals who had never smoked and had no history of substance abuse or dependence. Active smokers were classified as those who had smoked at least half a pack of cigarettes (i.e., 10 cigarettes) per day for over one year, based on the *Diagnostic and Statistical Manual of Mental Disorders, 4th Edition (DSM-IV)* criteria. Individuals who smoked fewer than 10 cigarettes per day were excluded. No participants had a history of alcohol abuse or psychiatric disorders.

The study was approved by the Institutional Review Board of Inner Mongolia Medical University and conducted in accordance with the *Declaration of Helsinki*. Written informed consent was obtained from all participants.

### 2.2 Assessments, biological sample collection, and laboratory analyses

Depressive symptoms were assessed using the Beck Depression Inventory (BDI). In this study, all participants had BDI total scores below 17, with higher scores indicating more severe depressive symptoms. The assessment was completed via self-report one day before cerebrospinal fluid (CSF) extraction.

Lumbar puncture is a standard clinical procedure in China for patients undergoing ACL reconstruction surgery, allowing convenient CSF collection while minimizing the potential influence of disease-related factors. Preoperative smoking cessation was not required for this procedure. A licensed anesthetist performed the lumbar puncture in the morning before surgery, administering 3 mL of 0.5% ropivacaine as local anesthesia. A 5-mL CSF sample was collected via lumbar puncture and immediately frozen at −80 °C. The ACL reconstruction procedure lasted less than one hour, and hospitalization before surgery did not exceed two days.

CSF concentrations of magnesium, zinc, iron, lead, lithium, copper, manganese, and aluminum were measured using atomic absorption spectrophotometry (29). Laboratory technicians were blinded to participants’ clinical data. Additional details on the assessment, biological sample collection, and laboratory tests conducted can be found in our previous study (23).

### 2.3 Statistical analysis

As described in our previous study, the normality of all variables was assessed using the Shapiro–Wilk test. Zinc, iron, and aluminum followed a normal distribution (p > 0.05). Consequently, the Mann–Whitney rank-sum test was used to compare demographic characteristics, clinical data, and biomarker levels between groups.

Spearman correlation was used to evaluate associations between CSF metal concentrations, BDI total scores and clinical features. All correlation p-values were adjusted via FDR correction for multiple comparisons, with the adjusted correlation matrix visualized. Traditional linear regression was employed to explore relationships between CSF concentration of metal ions and depressive symptoms for both smoker and non-smokers.

To assess the overall relationships among metal ions, clinical variables, and depressive symptoms, Mixed Graphical Models (MGM) were constructed using nodewise regression and EBIC-based model selection (R package mgm; parameters: k = 2, ruleReg = “OR”). Case-dropping bootstrap analysis (1,000 resamples, R package bootnet) was implemented to compute correlation stability (CS) coefficients for centrality stability evaluation (30). Network properties, including global structure, centrality indices, clustering coefficients, and centrality values, were analyzed to determine which of the CSF metal ion would emerge as central nodes within the CSF metal ion network accounting for the largest number of direct or indirect connections with depressive symptoms. Finally, moderation effect analysis was performed to further investigate how central nodes within the CSF metal ion network influenced the relationship between other variables and depressive symptoms.

All statistical analyses were conducted using *R* version 4.2.0. All tests were two-tailed, with a significance level set at p < 0.05.

## 3 Results

### 3.1 Sociodemographic and clinical characteristics

As shown in **Table 1**, active smokers had significantly higher Body Mass Index (BMI) (25.70 ± 3.40 vs. 24.37 ± 4.17, p = 0.01) and Beck Depression Inventory (BDI) total scores (3.65 ± 4.89 vs. 1.29 ± 2.15, p < 0.001) compared to non-smokers. Additionally, active smokers exhibited significantly higher cerebrospinal fluid (CSF) concentration of magnesium, zinc, iron, lead, lithium, and aluminum (all p ≤ 0.002).

**Table 1:** Comparisons between non-smokers and active smokers.

Variables	Non-smokers (n=104) (mean ± SD) (median, IQR)	Active smokers (n=57) (mean ± SD) (median, IQR)	p
Age (years)	29.61 ± 9.26 (28.00, 15)	32.49 ± 9.35 (31.00, 11)	0.05
Education (years)	13.04 ± 2.68 (15.00, 4)	12.05 ± 3.47 (15.00, 7)	0.12
BMI	24.37 ± 4.17 (23.58, 5.01)	25.70 ± 3.40 (25.51, 4.78)	0.01^*^
BDI total scores	1.29 ± 2.15 (0.00, 2.00)	3.65 ± 4.89 (2.00, 3.00)	<0.001^*^
Mg (mmol/L)	0.75 ± 0.48 (0.65, 0.40)	0.91 ± 0.20 (0.88, 0.23)	<0.001^*^
Zn (umol/L)	11.34 ± 1.40 (11.24, 1.95)	13.05 ± 1.49 (12.84, 2.25)	<0.001^*^
Fe (umol/L)	11.26 ± 1.63 (11.24, 2.25)	14.54 ± 1.23 (14.29, 1.64)	<0.001^*^
Pb (ug/L)	119.52 ± 17.36 (123.53, 18.91)	139.30 ± 13.02 (137.29, 18.21)	<0.001^*^
Li (mmol/L)	0.57 ± 0.17 (0.55, 0.27)	0.67 ± 0.12 (0.63, 0.16)	<0.001^*^
Cu (mg/L)	0.67 ± 0.12 (0.67, 0.12)	0.67 ± 0.08 (0.67, 0.12)	1.00
Mn (umol/L)	0.03 ± 0.01 (0.03, 0.01)	0.03 ± 0.01 (0.03, 0.01)	0.11
Al (umol/L)	0.88 ± 0.09 (0.88, 0.13)	0.93 ± 0.09 (0.93, 0.10)	0.002^*^
FTND	(--)	3.44 ± 2.27 (4.00, 3.50)	(--)
Age of smoking onset (years)	(--)	20.32 ± 4.25 (20.00, 4.00)	(--)
Smoking period (years)	(--)	12.23 ± 8.65 (10.00, 9.50)	(--)
Average number of cigarettes smoked per day	(--)	15.46 ± 7.31 (15.00, 10.00)	(--)

All data were reported as mean ± SD using Mann-Whitney sum tests and median with IQR. *p < 0.05.

BMI, body mass index (calculated as weight in kilograms divided by height in meters squared); BDI, Beck Depression Inventory; Mg, magnesium; Zn, zinc; Fe, iron; Pb, lead; Li, lithium; Cu, copper; Mn, manganese; Al, aluminum; FTND, Fagerstrom Test for Nicotine Dependence.

### 3.2 Correlation analysis

Spearman’s correlation analyses were performed to examine the relationships between CSF metal ion concentrations, BDI total scores, and clinical characteristics. False discovery rate (FDR) correction was applied to all correlation tests to adjust for multiple comparisons and reduce the risk of type I error, and the resulting correlation matrix is presented in **Figure 1**. Initially, no significant associations were observed between clinical features, such as Body Mass Index (BMI), and other variables. However, BDI total scores were positively correlated with the concentrations of four metal ions: zinc (Zn: r = 0.21, p < 0.05), iron (Fe: r = 0.21, p < 0.05), lead (Pb: r = 0.27, p < p < 0.05), and aluminum (Al: r = 0.28, p < 0.05).

**Figure 1:**
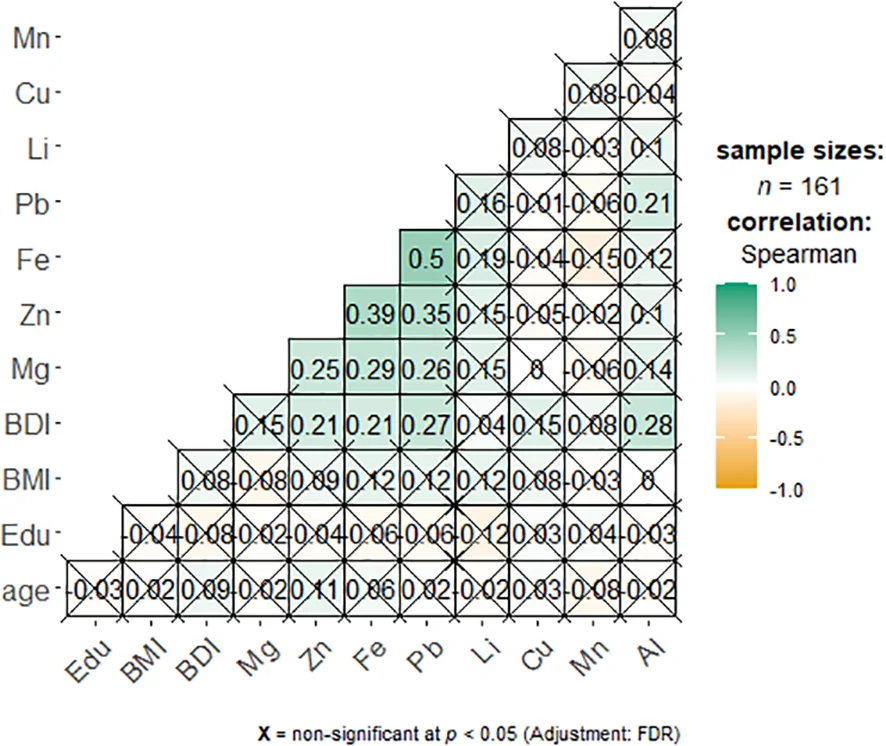
The correlation among demographic characteristics, metal ions, and BDI total scores. Partial correlation matrix adjusted for covariates, with false discovery rate (FDR) correction applied to control for type I error. Darker colors represent stronger correlations between variables (green colors = positive correlations, yellow colors = negative correlations). Statistical significance was determined based on FDR-adjusted *p* values. BMI, body mass index (calculated as weight in kilograms divided by height in meters squared); Edu, education; BDI, Beck Depression Inventory; Mg, magnesium; Zn, zinc; Fe, iron; Pb, lead; Li, lithium; Cu, copper; Mn, manganese.

These metal ions were not only related to depressive symptoms but also showed significant intercorrelations (Zn-Fe-Pb: all p < 0.01), suggesting that they may modulate depressive symptoms through a shared mechanism.

Further analysis of the relationship between Fe, Zn, Pb, and BDI total scores across different populations revealed that the association between these metal ions and BDI total scores was not significant when non-smokers and smokers were considered separately (for details see **Supplementary Figure 3**).

### 3.3 Network analysis

To further investigate the connections between various metal ions and Beck Depression Inventory (BDI) scores, we performed a network analysis using the mixed graphical model (MGM) framework. **Figure 2A** and **Supplementary Figure 1** illustrate the connections between metal ions and BDI total scores based on network edge weights. The network consists of 12 nodes, which include demographic information (age, BMI, education), BDI total scores, and CSF concentrations of eight metal ions. Of the 72 possible edges, 56 (61%) were set to zero, with an average edge weight of 0.036.

**Figure 2:**
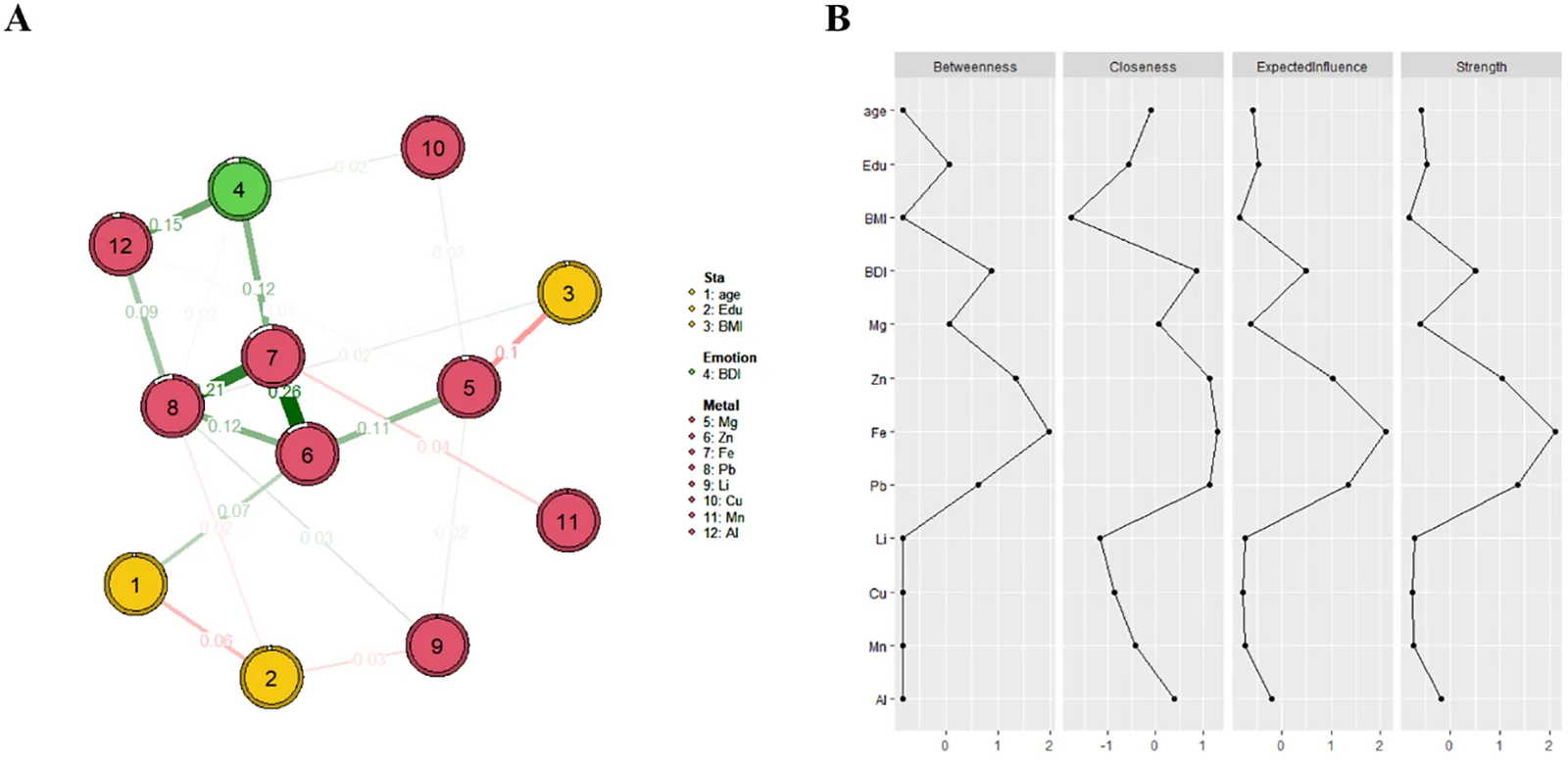
Network analysis of different metal ions and BDI total scores. **(A)** Network structure of different metal ions and BDI total scores, the thickness of the edge reflects the magnitude of the correlation, green edges represent positive correlations and red edges indicate negative correlations; **(B)** Centrality indices and Expected Influence of network. Standardized z-scores of strengths, closeness, betweenness and expected influence for each node in the network were plotted. Edu, education; BMI, body mass index (calculated as weight in kilograms divided by height in meters squared); BDI, Beck Depression Inventory; Mg, magnesium; Zn, zinc; Fe, iron; Pb, lead; Li, lithium; Cu, copper; Mn, manganese; Al, aluminum.

Initially, age, BMI, and education level (Edu) showed weak connections to the metal ion network and BDI total scores, exhibiting minimal association with the overall network (edge weight between BMI and Mg = 0.10; age and Zn = 0.07). In contrast, the relationships among metal ions were tightly interconnected, with 18.75% (6 out of 32) of the edges non-zero and an average edge weight of 0.05. Iron had the most connections with other metal ions. The strongest connections were observed between iron and zinc (edge weight = 0.26) and between iron and lead (edge weight = 0.21). Iron was an important node associated with BDI total scores (edge weight: Fe-BDI = 0.12). The network centrality indices for the metal ion network are shown in **Figure 2B**.

Iron, zinc, and lead ranked the highest in terms of strength and expected influence, indicating their central positions within the network. Specifically, the strength values were iron 2.10, lead 1.34, and zinc 1.03. These ions also exhibited the strongest potential for influence propagation, with expected influence values of 2.10 for iron, 1.34 for lead, and 1.03 for zinc. Notably, case-dropping bootstrapping demonstrated that strength and expected influence possessed satisfactory stability (CS-coefficient > 0.5), supporting robust interpretation of these indices (**Supplementary Table 1**). Although closeness values were iron 1.29, lead 1.14, and zinc 1.13, the closeness metric exhibited low stability (CS-coefficient < 0.25), and we therefore interpret closeness only in an exploratory manner without drawing definitive inferences based on this indicator. Similarly, bridge betweenness showed poor stability and should be considered exploratory (**Supplementary Table 1**). Furthermore, these three ions, along with BDI total scores, displayed elevated bridge betweenness scores (BDI = 0.89, Pb = 0.61, Fe = 1.97, Zn = 1.34); given insufficient stability of betweenness, we cautiously speculate that they may serve as connectors in the network, and conclusions relying solely on bridge betweenness should be treated with caution. Overall, the relationships among the metal ions are highly interconnected.

Iron and aluminum emerged as two of the most significant factors, collectively accounting for over 90% of the direct or indirect connections with depressive symptoms. The relationship between aluminum and depressive symptoms has been discussed in our previous study, and the results here further suggest that connections between aluminum and depressive symptoms is relatively independent within the metal ion network. In contrast, iron not only accounts for approximately 40% of the connections with depressive symptoms but also plays a crucial connecting role within the network (**Supplementary Figure 2**). The network analysis indicated that changes in CSF concentration of iron connected other metal ions to BDI total scores across the entire CSF metal ion network.

### 3.4 Moderation analysis

Moderation models were conducted based on the observed correlations (**Figure 1**) to explore whether metal ions moderate the relationship between smoking and BDI total scores. Initially, we assessed the impact of smoking on BDI total scores while controlling for age, education (Edu), and BMI. The linear regression analysis confirmed that smoking was positively associated with BDI total scores, suggesting that smokers tend to exhibit more severe depressive symptoms (β = 0.324, t = 4.129, p < 0.001; **Table 2**, Model 2).

**Table 2:** Linear regression table for the moderation analysis.

Variables	Model 1 (BDI)			Model 2 (BDI)			Model 3 (BDI)		
	β	t	95% CI	β	t	95% CI	β	t	95% CI
Age	0.010	0.132	-0.148-0.169	-0.033	-0.431	-0.186-0.119	-0.019	-0.249	-0.170-0.132
Edu	-0.07	-0.878	-0.229-0.088	-0.026	-0.341	-0.179-0.126	-0.049	-0.639	-0.200-0.102
BMI	0.022	0.282	0.134-0.179	-0.031	-0.403	-0.183- 0.121	-0.042	-0.547	-0.191- 0.108
Smoke	(--)	(--)	(--)	0.324 ^***^	4.129	0.169-0.479	0.068	0.498	-0.200-0.335
Fe	(--)	(--)	(--)	(--)	(--)	(--)	0.157	1.391	-0.066-0.379
Smoke: Fe	(--)	(--)	(--)	(--)	(--)	(--)	0.316^*^	2.563^*^	0.072-0.559
R²	-0.01			0.081^**^			0.110^***^		
F	0.3064 (3, 157)			4.516 (4, 156)			4.287 (6, 154)		

^*^ p<0.05, ^**^p<0.01, ^***^p<0.001.

Edu, education; BMI, body mass index (calculated as weight in kilograms divided by height in meters squared); BDI, Beck Depression Inventory; Fe, iron.

Next, we included CSF concentration of iron and the interaction term between smoking and iron. The regression analysis revealed that the interaction between smoking and iron showed the strongest association with depressive symptoms (β = 0.316, t = 2.563, p < 0.05; **Table 2**, Model 3). After incorporating the interaction term, the relationship between smoking, iron, and BDI total scores became non-significant, suggesting that the interaction term accounted for a substantial proportion of the observed association.

To better understand the interplay within the metal ion network and its impact on depressive symptoms, we examined the interaction effects of the four primary ions (iron, zinc, lead, and aluminum). The results revealed significant interaction effects between iron and zinc, as well as between iron and lead (Fe-Zn: β = 0.164, t = 2.124, p < 0.05; Fe-Pb: β = 0.232, t = 2.740, p < 0.01; **Table 3**).

**Table 3:** Linear regression table for the moderation analysis.

Variables	Model 1 (BDI)			Model 2 (BDI)			Model 3 (BDI)		
	β	t	95% CI	β	t	95% CI	β	t	95% CI
Age	-0.001	-0.014	-0.155-0.152	-0.022	-0.281	-0.175-0.131	0.004	-0.059	-0.155- 0.146
Edu	-0.049	-0.627	-0.203-0.105	-0.049	-0.631	-0.201-0.103	-0.02	-0.325	-0.176- 0.126
BMI	-0.009	-0.114	-0.162-0.144	-0.020	-0.255	-0.171-0.132	-0.048	-0.580	-0.196- 0.107
Fe	0.265^***^	3.393^***^	0.110-0.418	0.203^*^	2.392^*^	0.035-0.369	0.193^*^	2.201^*^	0.019-0.366
Zn	(--)	(--)	(--)	0.066	0.780	-0.101-0.234	(--)	(--)	(--)
Pb	(--)	(--)	(--)	(--)	(--)	(--)	0.071	0.801	-0.104-0.246
Fe: Zn	(--)	(--)	(--)	0.164^*^	2.124	0.011-0.316	(--)	(--)	(--)
Fe: Pb	(--)	(--)	(--)	(--)	(--)	(--)	0.232^**^	2.740^**^	0.064-0.400
R²	0.0504			0.073			0.125^**^		
F	3.123 (4, 156)			3.103 (6, 154)			3.554 (6, 154)		

^*^ p<0.05, ^**^p<0.01, ^***^p<0.001.

Edu, education; BMI, body mass index (calculated as weight in kilograms divided by height in meters squared); BDI, Beck Depression Inventory; Fe, iron; Zn, zinc; Pb, lead.

Furthermore, we tested the models including smoking and aluminum ions as covariates. The results showed that these interaction effects persisted even after considering the influence of smoking status and aluminum ions. The Fe-Zn interaction was marginally significant when accounting for aluminum ions (p = 0.06; **Supplementary Tables 2**–**4**).

## 4 Discussion

In this study, we examined the relationships among smoking status, CSF metal ion profiles, and depressive symptoms, with particular attention to the potential role of network structure and moderation effects. First, it was observed that smokers and non-smokers differed in the CSF concentration of various metal ions. Specifically, significantly higher CSF concentrations of magnesium, zinc, iron, lead, lithium, and aluminum were observed in smokers compared to non-smokers providing partial evidence that indicate smoking as a variable, could be used to explain differences and alterations in central metal homeostasis. Together with the observation that several CSF concentration metal ions were significantly associated with BDI total scores. The results depict how CSF concentrations of metal ions play a crucial role in reflecting the interrelationships between smoking and various degrees of reported depressive symptoms. Network analysis results further confirmed two of the eight metal ions, specifically iron and aluminum accounted for over 90% of the direct or indirect connections with BDI total score. Interestingly, unlike aluminum which is rather independent from other metal ions within the network. Significant interaction effects between iron and zinc, as well as iron and lead revealed iron as an important associated network node connecting various other metal ions and BDI total score. Additional moderation analysis identified a statistical interaction involving iron between smoking status and self-reported depressive symptoms. Together these findings shed light on how alterations in CSF concentration of metal ions, specifically iron, relate to various degrees of reported depressive symptoms and smoking.

It is well-established that cigarette smoke contains a wide range of metal constituents and can influence both direct exposure to exogenous metals and endogenous metal regulation (12, 22). In addition to inhalational exposure, chronic smoking has been speculated to increase oxidative stress, systemic inflammation, endothelial dysfunction, and changes in blood–brain barrier integrity, all of which may affect the transport, redistribution, and central availability of metal ions (12). Smoking is also reported to influence nutritional status and metabolic processes, which could further contribute to disturbances in metal homeostasis. The observed differences in CSF concentrations of metal ions between smokers and non-smokers extend upon previous findings based on peripheral specimens which reported of smoking-related metal dysregulation in the blood, urine, and hair. This finding indicates that smoking significantly changes not only peripheral metal ion profiles but also CSF metal ion profiles, and that such alterations is detectable in the CNS.

We also found that CSF concentration of several metal ions was associated with individual’s self-report of depressive symptom severity as measured by BDI total scores. This observation is consistent with the prevailing view that metal homeostasis is relevant to emotional regulation and neuropsychiatric function. Essential metals such as iron, zinc and magnesium are involved in multiple neurobiological processes related to depressive symptoms such as monoaminergic neurotransmission, synaptic plasticity, mitochondrial energy metabolism, myelination, and inflammatory regulation (31, 32). Excessive accumulation of potentially neurotoxic metals was reported to contribute to oxidative injury, neuroinflammation, and neuronal dysfunction (31–33). Although previous studies have reported associations between peripheral metal levels and depressive symptoms, the findings have been heterogeneous, likely due to differences in study populations, biological matrices, smoking exposure, nutritional status, comorbidities, and outcome definitions. Our results add to this literature by examining CSF concentration of metal ions, which may capture alterations of metal ions in the CNS. At the same time, because BDI total scores represent depressive symptom burden rather than a formal diagnostic classification, our findings may be more appropriately interpreted as reflecting symptom-related biological variation rather than disorder-specific pathophysiology.

A particularly notable finding was the role of iron in both the network and moderation analyses. In the network model, results indicate that CSF concentration of iron not only accounts for approximately 40% of the connections with self-reported severity of depressive symptoms. Moreover, it is an important associated network node connecting and bridging various other metal ions to depressive symptoms within the metal ion network in our analysis. In addition, the moderation analysis further revealed iron as a significant moderator which influenced the strength of the association between smoking and BDI total scores. These results are potentially important because they suggest that iron may not simply be one of many correlated metals, but rather a more informative component of the broader biological framework in which smoking and depressive symptoms are connected. Nevertheless, this interpretation should remain cautious. Network centrality does not establish causal priority, and a statistically significant interaction does not by itself identify a mechanistic pathway. Rather, these findings indicate that iron may represent a candidate marker, or a potentially relevant biological factor, that warrants further investigation.

The literature on iron further supports our results depicting iron as an important metal ion in the central nervous system. It is well-established in the literature that iron is required for enzymes involved in dopamine and serotonin metabolism, contributes to mitochondrial respiration and cellular energy production, and is essential for myelin formation and normal neuronal function (6–8, 34–36). Whereas, dysregulated iron metabolism may increase vulnerability to oxidative stress through redox-active reactions, thereby promoting neuroinflammation and neuronal injury (33, 37, 38). Both iron deficiency and iron excess has been reported to affect mood in a negative manner (34, 39), suggesting that iron-related effects on mood could be nonlinear and context dependent. Among smokers, these processes may be further modified by smoking-related oxidative burden, inflammatory activation, or altered metal transport. From this perspective, our findings support the view that disruption of central metal homeostasis in smokers is associated with depressive symptoms. However, the present data cannot determine whether altered iron levels are a cause, a consequence, or simply a correlate of depressive symptoms in smokers. Further study is needed to confirm that alteration of iron level via exogenous/endogenous means could influence the association between smoking and perceived of depressive symptoms.

From a clinical and translational perspective, these findings may have several implications. First, they suggest that self-report assessment of depressive symptoms perceived and experienced by male smokers may not be explained solely by psychosocial or behavioral factors, but may also involve measurable biological alterations in the central microenvironment. Second, the results and findings provide a glimpse of the possibility that metal-related indicators could help refine biological stratification in smoking populations with depressive symptoms, particularly if future studies can identify peripheral proxies that correlate with CSF findings. Third, the prominent correlation observed for iron can provide clues for subsequent longitudinal and experimental research. Future studies may further explore whether smoking-induced disturbances of iron homeostasis correlate with depressive symptoms via oxidative stress, inflammatory responses, or neurotransmitter-related biological correlates. However, the clinical applicability of these findings remains limited at present. CSF sampling is invasive and not suitable for routine screening, and our results do not support immediate therapeutic interventions targeting metal homeostasis without further validation.

This study also has several strengths. By analyzing CSF rather than relying exclusively on peripheral specimens, we were able to assess a biologically relevant compartment that is more closely related to the central nervous system. In addition, we examined smoking, depressive symptoms, and a panel of metal ions within a unified analytical framework, and further incorporated network and moderation approaches to move beyond conventional pairwise associations (40, 41). These features allowed us to generate a more integrated view of the potential relationships among smoking exposure, central metal homeostasis, and depressive symptoms.

Future studies should aim to validate these findings in larger and more diverse cohorts where male and female smokers are present, ideally using longitudinal designs to clarify the temporal sequence of smoking exposure, metal dysregulation, and depressive symptom development. Studies combining CSF and peripheral measurements may also be useful for identifying clinically feasible surrogate indicators of central metal homeostasis. In addition, mechanistic work is needed to determine whether iron-related pathways, including inflammation, mitochondrial dysfunction, inflammatory signaling, and neurotransmitter metabolism, contribute to the observed associations. It would also be valuable to examine whether smoking cessation is accompanied by normalization of metal ion profiles and improvement in depressive symptoms, which could help clarify the clinical relevance of these biological alterations.

## 5 Limitations

Despite revealing CSF concentrations of iron interaction with other metal ions and how iron levels influenced the strength of the association between smoking and self-reports of depressive symptoms, this study has several limitations. First, the participants were patients with anterior cruciate ligament injuries, which may affect the generalizability of our results compared to those obtained from healthy individuals. Second, due to the relatively low number of female smokers in China, only male participants were recruited, which highlights the need for future studies to explore gender differences in depressive symptoms. Third, depressive symptoms were assessed using self-report BDI scores rather than a structured psychiatric diagnosis, so the results may not generalize directly to clinically diagnosed major depressive disorder. Fourth, all analyses were performed using total BDI total scores, so we could not re-analyze data by removing somatic items. Somatic discomfort induced by injury, pain, inflammation and metal exposure cannot be differentiated from depressive cognitive-affective symptoms. Additionally, only age, education and BMI were adjusted as covariates, leaving potential confounders such as pain severity, diet and occupational metal exposure unaccounted for when interpreting metal-BDI associations. Fifth, cadmium was not included in the prespecified CSF metal panel used for the present analysis limiting the present study’s ability to investigate how cadmium may influence the findings of this study. Sixth, indicators reflecting the level of smoking exposure (e.g., FTND score) were not incorporated into our main analytical models. Larger cohorts are warranted in future research to systematically examine the dose-response relationships between smoking exposure, metal ion concentrations, and depressive symptoms. Future study should also include peripheral blood and urinary metal data to obtain a better understanding of peripheral metal burden to obtain a holistic understanding of metal-smoking-depressive symptom relationship.

## 6 Conclusion

In conclusion, our findings suggest that smoking is associated with alterations in selected CSF metal ions and that these alterations may be related to depressive symptoms in active male smokers. Iron was identified as an important associated node connecting various other metal ions and BDI total score, supporting the relevance of central metal dysregulation to the smoking–depressive symptoms in active male smokers.

## Data Availability

The raw data supporting the conclusions of this article will be made available by the authors, without undue reservation.

## Supplementary material

Supplementary Table 1 Bootstrap stability coefficients and threshold test results of centrality indices.

Supplementary Table 2 Linear regression table for the moderation analysis. cp<0.05, **p<0.01, ***p<0.001. Edu, education; BMI, body mass index (calculated as weight in kilograms divided by height in meters squared); Al, aluminum; Fe, iron.

Supplementary Table 3 Linear regression table for the moderation analysis. *p<0.05, **p<0.01, ***p<0.001. Edu, education; BMI, body mass index (calculated as weight in kilograms divided by height in meters squared); BDI, Beck Depression Inventory; Fe, iron; Zn, zinc.

Supplementary Table 4 Linear regression table for the moderation analysis. *p<0.05, **p<0.01, ***p<0.001. Edu, education; BMI, body mass index (calculated as weight in kilograms divided by height in meters squared); BDI, Beck Depression Inventory; Fe, iron; Pb, lead.

Supplementary Figure 1 Centrality and bridge centrality indexes of network.

Supplementary Figure 2 Edge stability test for edge weights and centrality indices. **(A)** Edge stability test bootstrap estimates 95% confidence interval for edge weights; **(B)** Edge stability test bootstrap estimates 95% confidence interval for centrality indices.

Supplementary Figure 3 Linear regression associations of Fe, Zn, and Pb with BDI total scores stratified by smoking status (class 0: non-smokers; class 1: active smokers). **(A)** Iron; **(B)** Zinc; **(C)** Lead. BDI, Beck Depression Inventory; Fe, iron; Zn, zinc; Pb, lead.
